# Parity-Time symmetry helps breaking a new limit

**DOI:** 10.1038/s41377-024-01577-0

**Published:** 2024-09-19

**Authors:** Wenjie Wan, Xiaoshun Jiang

**Affiliations:** 1 University of Michigan-Shanghai Jiao Tong University Joint Institute, Shanghai Jiao Tong University, Shanghai, 200240 China; 2 National Laboratory of Solid State Microstructures and College of Engineering and Applied Science, Nanjing University, Nanjing, 210093 China

**Keywords:** Microresonators, Nonlinear optics

## Abstract

Parity-Time (PT) symmetry is an emerging concept in quantum mechanics where non-Hermitian Hamiltonians can exhibit real eigenvalues. Now, PT symmetric optical microresonators have been demonstrated to break the bandwidth-efficiency limit for nonlinear optical signal processing.

## Abstract (web summary)

Parity-Time symmetric micro-resonators utilizing nonlinear four-wave mixing, now surpass the bandwidth-efficiency limit for optical signal processing, achieving a remarkable 100-fold efficiency boost.

## Graphical abstract

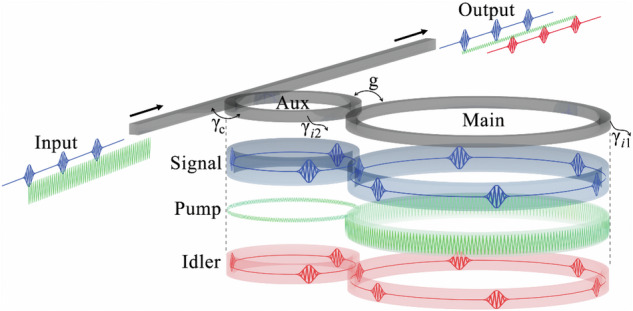

Parity-Time (PT) symmetry has emerged as a key concept in quantum mechanics, allowing non-Hermitian Hamiltonians to exhibit entirely real eigenvalues under specific conditions^1^. This symmetry plays a crucial role in systems where balanced absorption and amplification coexist, leading to a phase transition called PT-symmetry breaking and the emergence of complex eigenvalues beyond a critical threshold. PT symmetry has significant implications in optics and photonics, particularly in areas such as waveguides^2^, microresonators^3,4^ and lasers^5^. Practical applications of PT symmetry include loss-induced transparency^2^, unidirectional invisibility^6^, and enhanced sensing capabilities^7,8^. The controllable manipulation of gain and loss in PT-symmetric systems has opened up new possibilities for advanced signal processing, communication technologies, and optical devices, paving the way for innovative solutions across various disiplines.

Recently, in a newly published paper in eLight^9^, Jing Xu from Huazhong University of Science and Technology, China and Minhao Pu from Technical University of Denmark, have jointly demonstrated a PT-symmetric microresonator system. This innovative system not only enhances light intensity but also enables high-speed operation, overcoming the limitations of conventional setups based on single resonators. By combining PT symmetry with near-exceptional point operation^10^, a new nonlinear optical signal processing (NOSP) system utilizing four-wave mixing (Fig. 1) achieves a remarkable two-orders-of-magnitude improvement in efficiency. Using a highly nonlinear AlGaAs-on-Insulator platform, NOSP at nearly 40 gigabits per second is demonstrated with a remarkably low pump power of one milliwatt.

**Fig. 1:**
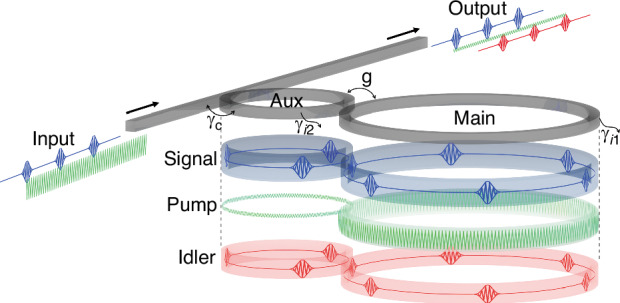
Schematic the PT-symmetric coupled microresonators for ultrafast nonlinear optical signal processing. The intracavity field distribution of signal, pump, and idler wave indicated by blue, green, and red colored shades, respectively. The signal and idler wave pulses in the cavities depict high-speed data-encoded signal and idler waves

The breakthrough addressed a critical challenge in implementing nonlinear optical signal processing, which demands high-intensity light fields. While NOSP shows promise for enhancing optical communication networks with ultrafast processing speeds and improved efficiency, generating and maintaining the necessary high-intensity light fields has posed a significant challenge. This obstacle has hindered the practical realization of NOSP systems for high-speed, high-capacity optical communications. These results pave the way for fully chip-scale NOSP devices with integrated pump laser, promising applications in optical communication networks and classical or quantum computation. Furthermore, the synergy between PT symmetry and NOSP presents new opportunities in amplification, detection, and sensing, addressing the need for both speed and efficiency.

## References

[1] Bender, C. M. & Boettcher, S. Real spectra in non-hermitian hamiltonians having symmetry. *Phys. Rev. Lett.* **80**, 5243–5246 (1998).

[2] Guo, A. et al. Observation of -Symmetry breaking in complex optical potentials. *Phys. Rev. Lett.* **103**, 093902 (2009). 19792798 10.1103/PhysRevLett.103.093902

[3] Chang, L. et al. Parity–time symmetry and variable optical isolation in active–passive-coupled microresonators. *Nat. Photonics* **8**, 524–529 (2014).

[4] Peng, B. et al. Parity–time-symmetric whispering-gallery microcavities. *Nat. Phys.* **10**, 394–398 (2014).

[5] Feng, L. et al. Single-mode laser by parity-time symmetry breaking. *Science* **346**, 972–975 (2014). 25414307 10.1126/science.1258479

[6] Lin, Z. et al. Unidirectional invisibility induced by -Symmetric periodic structures. *Phys. Rev. Lett.* **106**, 213901 (2011). 21699297 10.1103/PhysRevLett.106.213901

[7] Chen, W. J. et al. Exceptional points enhance sensing in an optical microcavity. *Nature* **548**, 192–196 (2017). 28796206 10.1038/nature23281

[8] Lai, Y. H. et al. Observation of the exceptional-point-enhanced Sagnac effect. *Nature* **576**, 65–69 (2019). 31802018 10.1038/s41586-019-1777-z

[9] Kim, C. et al. Parity-time symmetry enabled ultra-efficient nonlinear optical signal processing. *eLight* **4**, 6 (2024). 38585278 10.1186/s43593-024-00062-w PMC10995095

[10] Chen, Y. et al. Exceptional points with memory in a microcavity Brillouin laser. *Optica* **9**, 971–979 (2022).

